# Computer‐Aided High Tibial Osteotomy—A Comparative Study of Commonly Used 3D Printing Technology and Navigation Application

**DOI:** 10.1111/os.14274

**Published:** 2024-12-23

**Authors:** Elvis Chun‐Sing Chui, Kyle Ka‐Kwan Mak, Randy Hin‐Ting Ng, Ericsson Chun‐Hai Fung, Mei‐Shuen Chan, Kai Yue, Lawrence Chun‐Man Lau, Clifford Long‐Fung Chan, Edmond Wing‐Fung Yau, Wei Zhao, Xiuyun Su, Jin Zhang, Jianglong Xu, Hongxun Sang, Guoxian Pei, Louis Wing‐Hoi Cheung, Sheung‐Wai Law, Michael Tim‐Yun Ong, Patrick Shu‐Hang Yung

**Affiliations:** ^1^ Department of Orthopaedics and Traumatology The Chinese University of Hong Kong Hong Kong China; ^2^ Department of Orthopaedics and Traumatology Prince of Wales Hospital Hong Kong China; ^3^ Koln 3D Technology Medical Ltd Hong Kong China; ^4^ Department of Orthopaedics Southern University of Science and Technology Hospital Shenzhen China; ^5^ Department of Orthopaedics Shenzhen Hospital of Southern Medical University Shenzhen China; ^6^ Department of Orthopaedics Shenzhen Children's Hospital Shenzhen China

**Keywords:** 3D printing, direct metal laser sintering, fused deposition modeling, high tibial osteotomy, navigation system, selective laser sintering

## Abstract

**Background:**

High tibial osteotomy (HTO) is a surgical procedure for treating certain knee conditions. Proper execution of HTO can preserve joint function and delay or avoid the need for total knee replacement. This study compared different 3D printing techniques (fused deposition modeling, selective laser sintering, and direct metal laser sintering) and a navigation system for their suitability in assisting HTO surgeries.

**Methods:**

Tibial saw‐bones were used as models, and surgical guides and the navigation system were employed during the procedures. Six parameters (planning time, manufacturing time, delivery time, material cost, operation time, and accuracy) were evaluated. One‐way analysis of variance (ANOVA) and *t*‐test were used for the analysis.

**Results:**

The results showed that the metal surgical guides had the highest accuracy (angle differences mean, 2.4°) and operation time (mean 9.75 min), followed by plastic guides, classic guides, and the navigation system. The differences in accuracy were attributed to factors like rigidity, melting point, and errors during incisions.

**Conclusions:**

The study recommended metal surgical guides as the best option for assisting HTO due to their accuracy and operation time. And the results have implications for orthopedic surgeons performing HTO surgeries, as they can use this information to improve postoperative outcomes, such as mechanical axis alignment and quality of life for HTO patients.

## Graphical abstract

This outlines the structured workflow of an experimental study designed to evaluate and compare the efficacy of printed surgical guides and navigation systems in High Tibial Osteotomy (HTO) surgery. The process involves preoperative imaging, detailed cut planning using specialized software, inputting the surgical plan into the navigation system, and 3D printing surgical guides from various materials based on the planned cuts. After the surgical procedures are performed, thorough postoperative assessments are conducted, emphasizing key outcome measures such as surgical precision.

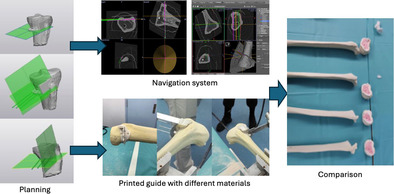

## 1 Introduction

High tibial osteotomy (HTO) is a surgical procedure performed to realign the knee joint in patients with medial compartment knee osteoarthritis. The goal of HTO is to shift the weight‐bearing forces from the damaged, worn‐out medial compartment to the relatively healthier lateral compartment of the knee joint. The procedure typically involves making a controlled cut in the proximal tibia (upper shin bone) and then either opening or closing the angle of the bone to shift the weight‐bearing forces. This is usually done through an open incision on the inside of the knee, but less invasive techniques using small incisions and bone cuts have also been developed [1, 2]. Overall, HTO is a valuable treatment option for selected patients with medial compartment knee osteoarthritis, allowing them to maintain an active lifestyle for a period before possibly needing a total knee replacement in the future.

3D printing has become a commonly used approach in orthopedic surgery [3, 4]. As technology has advanced, a variety of 3D printing materials have become available, including plastics, polymers, and metals. Fused deposition modeling (FDM) utilizes rigid plastic materials like ABS and nylon PA12 [5]. Selective laser sintering (SLS) uses powdered polymers like TPU, TPE, and polyamides [6]. Direct metal laser sintering (DMLS) can print with a range of metals and metal alloys, from stainless steel to aluminum [7]. These 3D printing materials have properties that make them suitable for manufacturing surgical guides, such as being lightweight, flexible, durable, and having desirable thermal characteristics [8, 9].

In addition to surgical guides, navigation systems can also be a useful tool for assisting orthopedic surgeries. There are several advantages to using navigation systems for HTO procedures. First, short planning time—Navigation systems do not require the design and manufacturing of surgical guides, so the planning phase is quicker. Second, more precise correction angles—navigation systems provide real‐time intraoperative data on the coronal, sagittal, and transverse axes, as well as the status of the medial and lateral soft tissue. This enables more accurate osteotomy angle corrections compared with traditional preoperative planning. And avoidance of unintended posterior tibial slope changes—the navigation system helps control the cortical hinge position during opening or closing wedge HTOs, preventing medial tibial collapse and unsuccessful procedures. Besides, higher accuracy and lower variability—studies have shown that HTO procedures performed with a navigation system resulted in better alignment of the mechanical axis through the tibial plateau, compared with the conventional cable method [10, 11]. Similarly, navigation systems have been shown to improve component placement accuracy in total hip arthroplasty (THA) procedures and help restore proper leg length. Overall, the use of navigation systems can provide significant benefits over traditional surgical approaches for orthopedic procedures like HTO [12].

This study aims to compare the differences between surgical guides made using three different 3D‐printing techniques in the context of preclinical HTO trials. Specifically, the study will focus on the following six different parameters: planning time, manufacturing time, material cost, delivery time, operation time, and accuracy.

## 2 Materials and Methodology

Since there were 4 conditions involved—metal, plastic, metal surgical guides, and navigation system, 10 saw‐bones were utilized in each of them. Surgical guides with the same structural design were used. Each guide consisted of two incision sites that mimicked two tunnels that were perpendicular to each other.

During the preoperative planning phase, the manufacturing procedure of the surgical guides was as follows: At first, 3D scanner (Shining Einscan Pro 2×) was used to scan the sawbones and output a stl file. The stl file was imported into 3‐Matic Software (Materalise, Belgium) where designing process of surgical guides was carried out. At last, the surgical guides were manufactured by either one of the above 3D printing techniques.

On the contrary, no surgical guides were needed for navigation‐assisted HTO but a number of steps were needed—(1) scanning of tibial sawbones by Mass 3D scan, (2) Mimics segmentation, (3) design of the cutting plane and introduction of six screws besides the cutting plane in 3‐Matic Software (Materialize, Belgium) and output of stl files, (4) import of cutting plane onto DICOM files containing the tibial sawbones and coloring of the cutting plane using Mimics Software (Materialize, Belgium) before the DICOM files containing the sawbones mounted with cutting plane and the screws (“modified” DICOM files) was uploaded to the “OrthoMap Module” of the navigation system (Stryker eNlite Navigation system).

### 2.1 Mars‐CT Scan Specification

**FIGURE 1:**
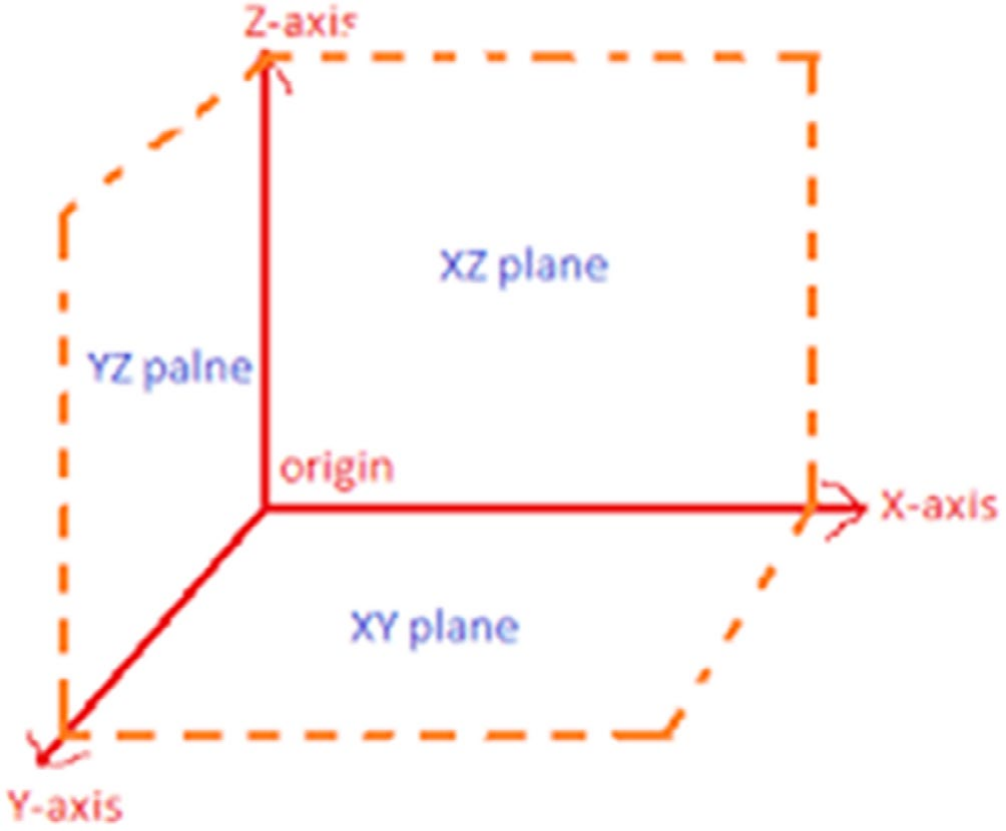
Display of world coordinate system. It illustrated three axes (X, Y, and Z axis) which formed the XZ‐plane, YZ‐plane, and XY‐plane. In this study, cuts were made in XY‐plane whereas the angle differences (which would be discussed in later section) were observed in XZ‐ and YZ‐planes.

**FIGURE 2:**
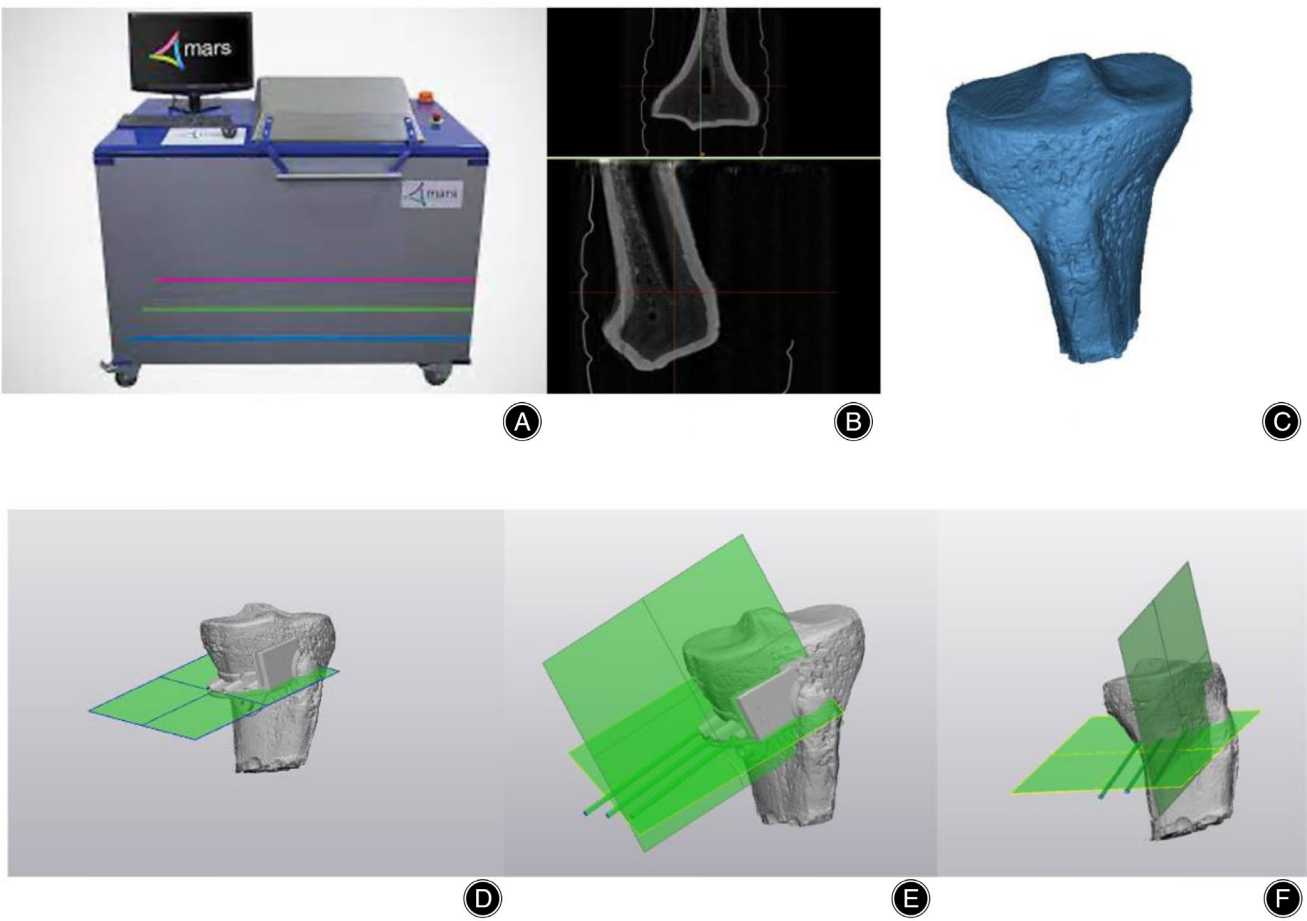
(A,B) The Mars CT scan machine and image in Mars CT, respectively. (C) The segmented 3D model. (D) The cutting‐plane planning. (E) Demonstrates the screw planning. (F) The final output of two cutting planes without the Jig.

**FIGURE 3:**
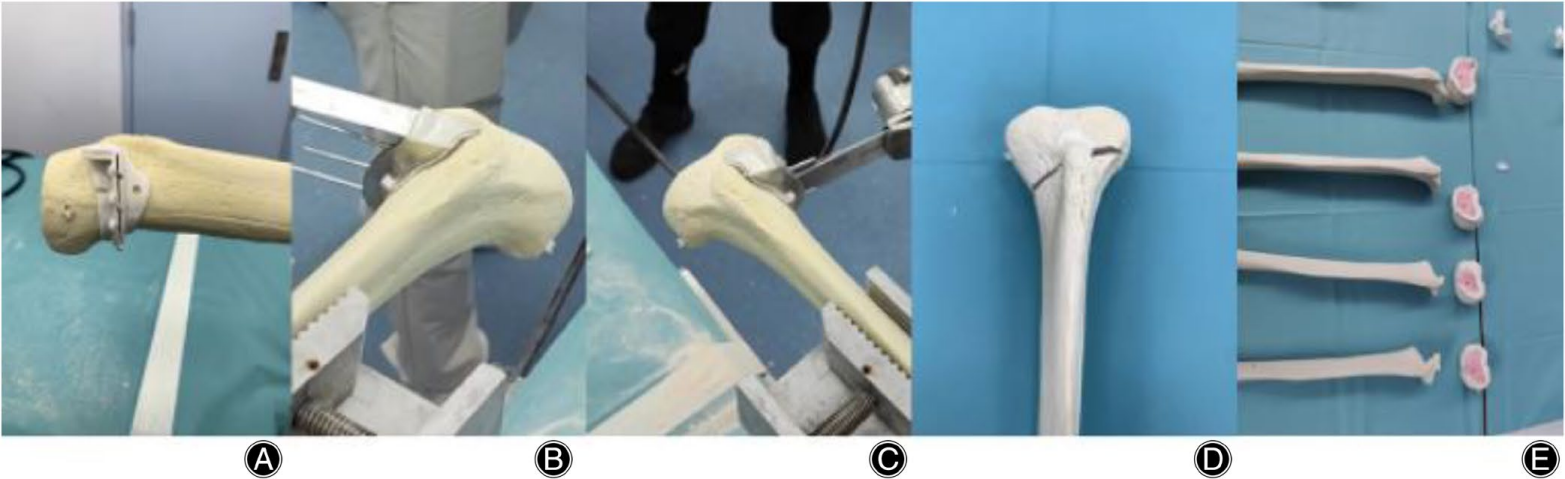
Illustration of Surgical Guide‐Assisted High Tibial Osteotomy (HTO). (A) The surgical guide is positioned on the bone model. (B) The K‐wire is inserted into the bone with the assistance of the guide. (C) The bone model is sectioned using a surgical blade. (D) Display of the cut bone model. (E) Visualization of the internal structure and the cutting plane of the bone model.

Traditional CT and x‐ray scan could not provide a clear image of the sawbone, the team decided to scan it with a spectral CT, called MARS CT from MARS Bioimaging Ltd. The MARS CT is equipped with photon detectors that can measure up to 8 energy bins per pixel, which allows the CT to obtain clear images of low‐energy materials. After the scan, a web‐based surgical platform (Artificial Intelligence Orthopedics Surgery Planning Platform) was used to perform HTO planning. The planned module was then imported into 3Matic for jig modeling. The jig model was 3D printed into surgical guide in three different materials, while the sawbone model was imported into the Stryker for navigation used. When it came to the “cutting” procedure, surgical saws were employed to perform two cuts (main cut and side cut) in XY‐axis at the two incision sites indicated by surgical guides. Since three axes will be mentioned in the paper, the coordinate system could be visualized in Figure 1. Main cut was where surgeons cut through the vertical narrow tunnel in surgical guide whereas side cut was made along the horizontal tunnel within the surgical guide (Figure 2). The undergoing of surgical guides‐assisted HTO differed from that of Navigation‐assisted HTO. Surgical guide‐assisted HTO was where the surgical guides were placed on the proximal region of the tibial sawbones and then screws of 2 mm diameter were inserted into the three holes of the guide for fixation purposes. Two types of cuts (main cut and side cut) were then made along the incision sites indicated by the surgical guides until the proximal tibial bone was removed. The steps of surgical guide‐assisted HTO would be demonstrated in Figure 3.

### 2.2 Assessment Parameters

**FIGURE 4:**
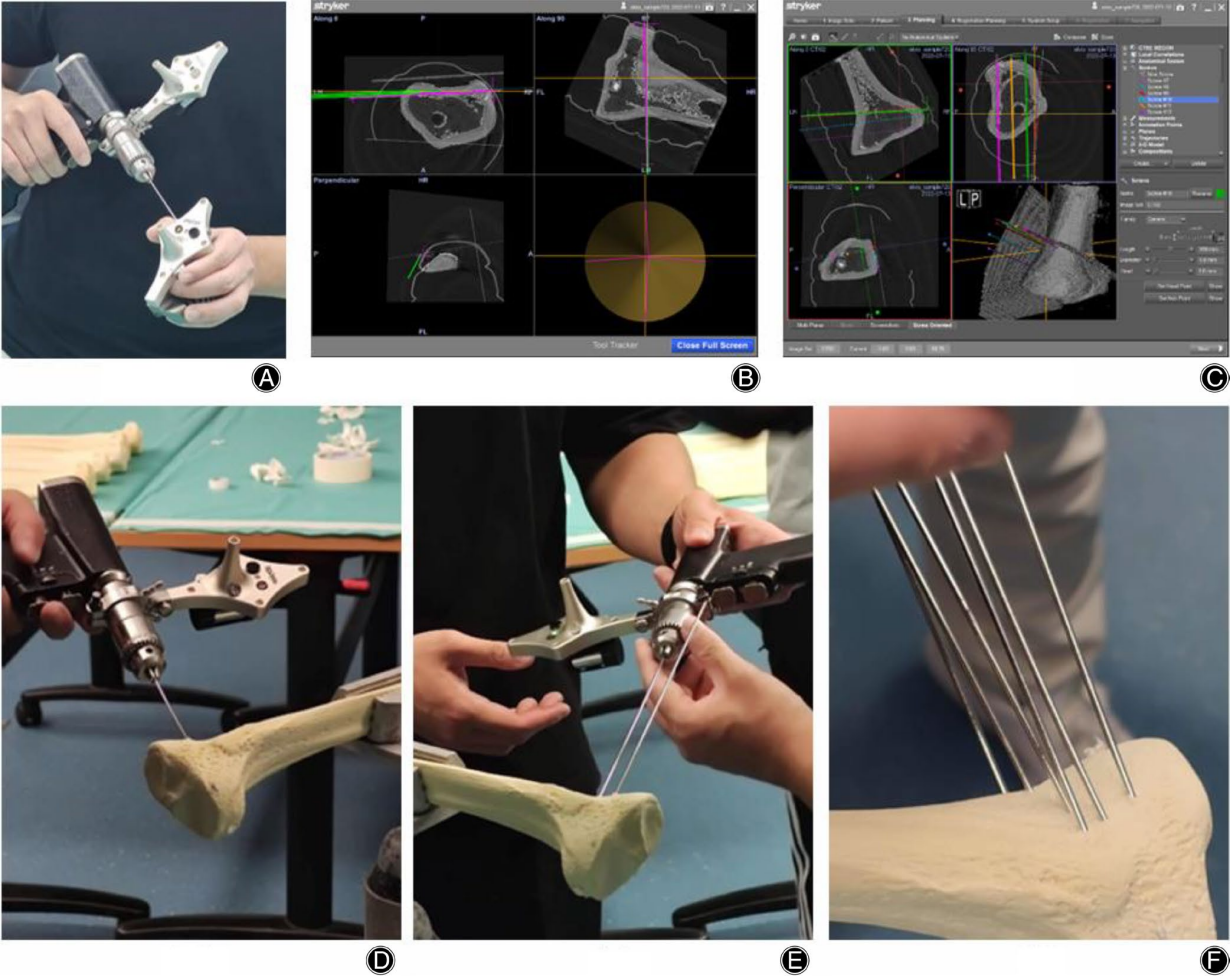
Illustration of Navigation‐Assisted High Tibial Osteotomy (HTO). (A) Calibration of the navigation system is performed. (B) User Interface (UI) of the navigation system during the insertion of the K‐wire. (C) UI of the navigation system displaying all inserted K‐wires. (D) and (E) Demonstration of the K‐wire insertion procedure. (F) Overview of the complete insertion of all K‐wires.

Numerous sophisticated steps were taken with the navigation system. As aforementioned, “modified” DICOM files were uploaded in the specific module of navigation system where five points matching and surface matching were undergone. This enabled the addition of six screws in geographical location (with respect to the two incision sites of surgical guide; three screws along each incision site) of the bones and two cuts (main cut and side cut) were then made along these screws. The steps of Navigation‐assisted HTO would be clearly demonstrated in Figure 4. At the beginning, the calibration and matching procedure of the navigation system would be done. With the aid of the navigation system on the screen, the screws/pins were inserted onto the proximal tibia region to form the site of main cut and side cut.

Throughout the trials, six parameters (planning time, operation time, manufacturing time, delivery time, material cost, and accuracy) were assessed. Planning time was the amount of time used for designing and printing of surgical guides or for designing of cutting plane and screw insertion in DICOM images. Operation time was the amount of time needed to perform HTO. Manufacturing time was the amount of time needed to generate the patient‐specific surgical guides. Delivery time was the amount of time needed to deliver the surgical guides. Material cost was measured per kilogram (kg) of the material needed to generate the surgical guides with different materials. Accuracy was assessed by the observation and calculation of angle difference between expected cuts made by the preoperatively planned cutting plane and actual cuts made according to the preplanned cutting plane in two axes (XZ‐ and YZ‐axis). Among all parameters, planning time and accuracy were measured 10 times in each condition.

### 2.3 Statistical Analysis

All data were analyzed using an unpaired using SPSS Version 26 (IBM, NY, USA). Data were expressed as mean ± standard deviation. One‐way analysis of variance (ANOVA) was used for the analysis of planning time, manufacturing time, delivery time, material cost, operation time, and accuracy, followed by *t*‐test for significant pairwise differences between subsamples. Results were considered as statistically significant when *p* < 0.05, unless otherwise specified.

## 3 Results

### 3.1 Overview of Surgical Planning and Execution

In this section, we break down the various aspects of preoperative planning, manufacturing times, operation durations, and accuracy of different surgical methods. The planning time for using a navigation system was notably efficient, requiring only 30 ± 0.5 min for preoperative planning. In contrast, the planning for surgical guides was more time‐consuming, taking 1.25 h. This significant difference highlights the quicker setup offered by the navigation system. When examining manufacturing times, surgical guides were produced in the following order of efficiency: metal > plastic > classic. Metal guides not only had the shortest manufacturing time but also ranked highest in delivery times, followed by plastic and classic guides, which were on par. This trend reflects the advanced processing capabilities of metal materials. Material costs also varied significantly among the different types of surgical guides. The ranking in terms of expense was as follows: metal (most expensive), plastic, classic (least expensive); this order suggests that while metal offers advantages in other areas, it comes at a higher financial cost.

In terms of operational efficiency, the navigation system required 31 ± 0.5 min for the positioning and insertion of screws, along with the osteotomy process. Conversely, using surgical guides streamlined the process, allowing for an average of only 10 min for HTO. This efficiency showcases the potential time savings when using surgical guides. Finally, when it comes to accuracy, the methods ranked as follows from highest to lowest: metal > plastic > classic > navigation system. This ranking indicates that while navigation systems may offer speed, they do not match the precision achieved with metal guides.

### 3.2 Visual Data Representation

**FIGURE 5:**
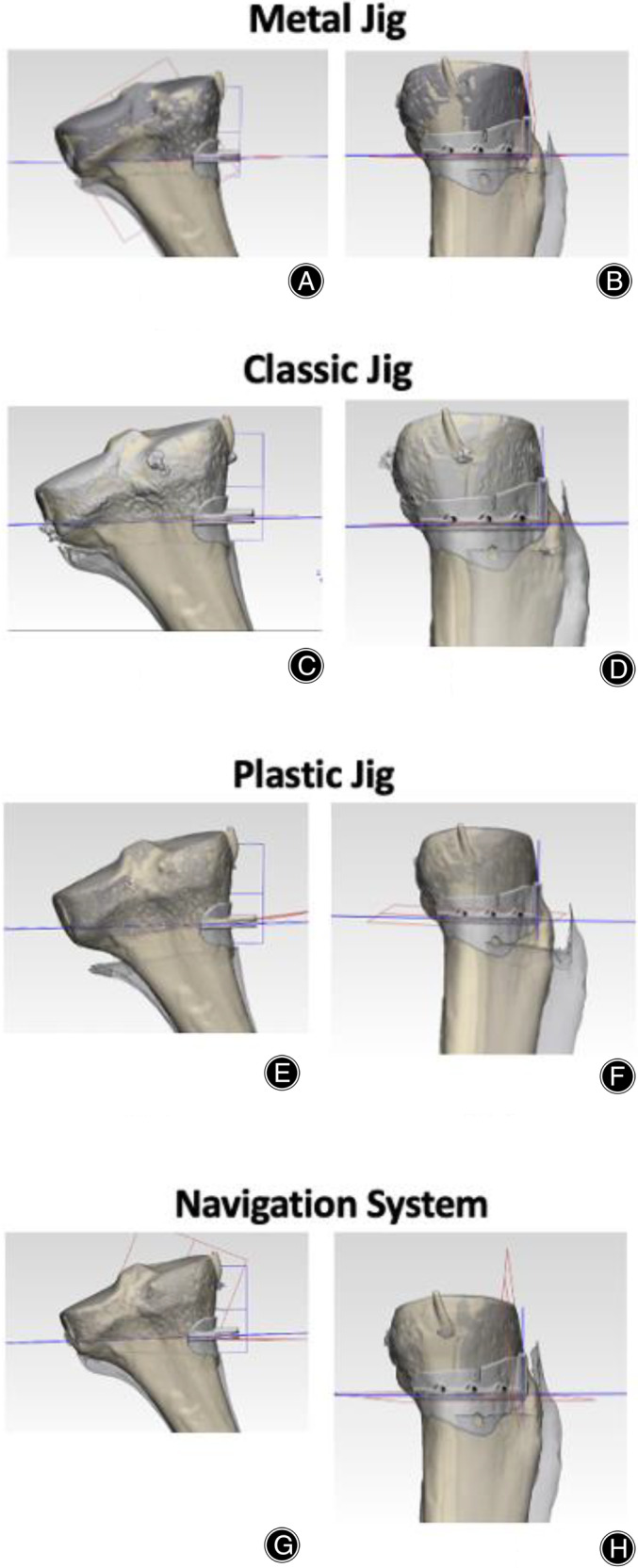
Illustration of angle differences in XZ‐ and YZ‐planes. Blue plane is the preplanned cutting plane whereas red plane is the actual cutting plane. (A,B) The angle difference when metal jig was used. (C,D) The angle difference when classic jig was used. (E,F) The angle difference when plastic jig was used. (G,H) The angle difference when navigation system was used.

**FIGURE 6:**
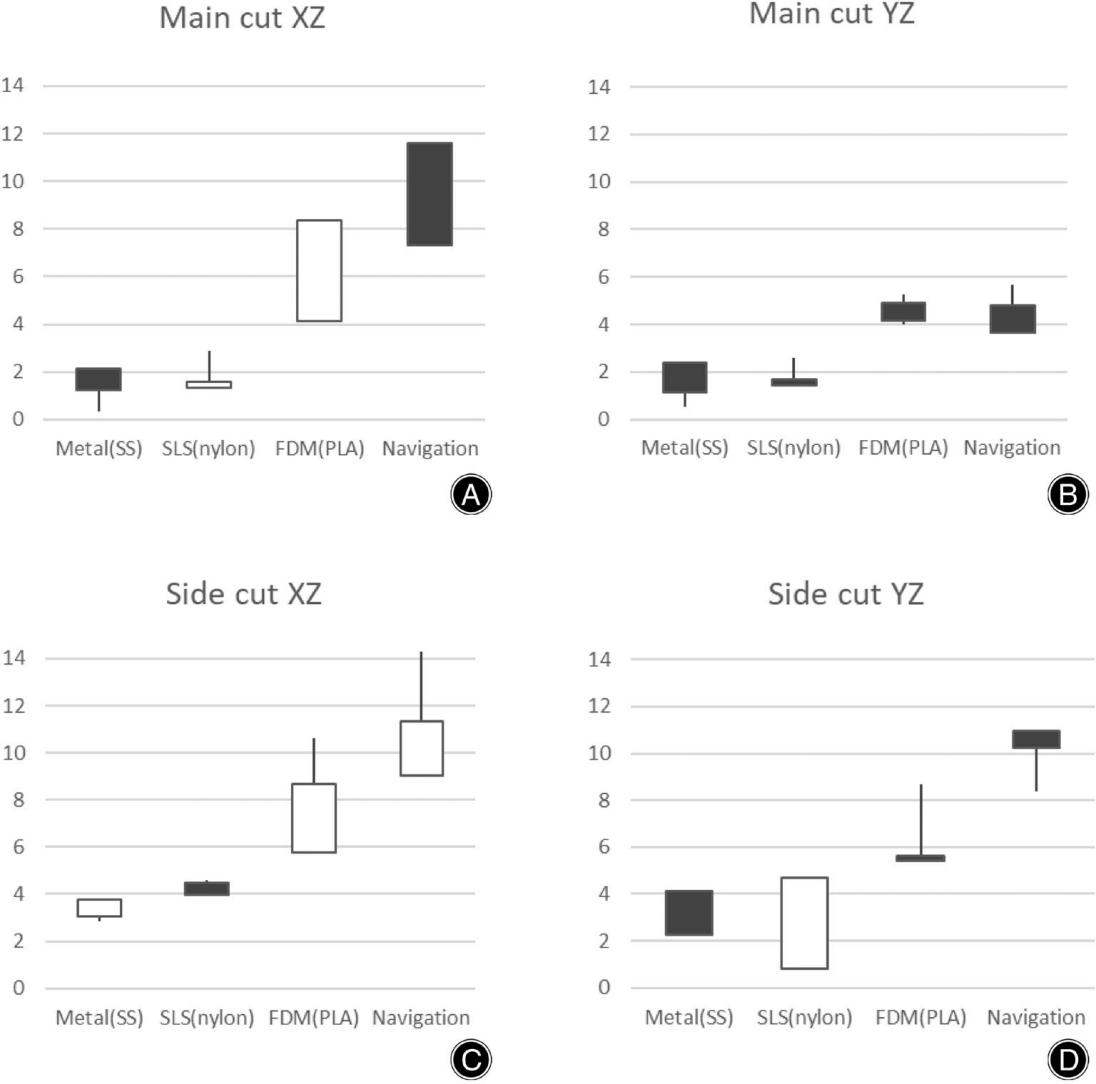
Demonstration of angle differences of three different 3D‐printed surgical guides and navigation system. The X‐axis displays the items under investigation (three different surgical guides and Navigation) whereas Y‐axis represents angle difference (i.e., the lower the value, the higher the accuracy of incision it was). (A,B) The angle differences during main cut (vertical cut) whereas (C,D) Angle differences during the side cut (horizontal cut).

For a clearer understanding of the data, Table 1 provides a comprehensive overview of each parameter, excluding accuracy. Additionally, Figure 5 displays the angle differences between the preoperative planned cutting plane and the actual intraoperative cutting plane across the four different conditions. To further elucidate these angle differences, Figure 6 features box‐and‐whisker plots that visually represent the variations in outcomes depending on the surgical method used.

**TABLE 1:** Illustration of six parameters in four different conditions.

	Metal 3D printing	Plastic 3D printing	Classic 3D printing	Navigation system
Preoperative planning time (average) (mins)	73.4	76	75.6	30.4
Manufacturing time	7 days	1 day	4 h–1 day	NA
Delivery time (business days)	12	6	6	NA
Material cost (USD) per kilogram	150	135	30	NA
Operation time (mins)	9.75	10.25	10	31.5
Average angle differences (degrees)	2.40	2.95	6.60	8.84

Abbreviation: NA, non‐applicable.

## 4 Discussion

HTO is a well‐established surgical procedure primarily aimed at correcting knee malalignment, particularly in patients with medial compartment osteoarthritis. The advent of computer‐aided technologies, specifically 3D printing and navigation applications, has introduced new paradigms in the planning and execution of HTO. The study recommended metal surgical guides as the best option for assisting HTO due to their accuracy and operation time.

### 4.1 Manufacturing Time

According to the findings, the manufacturing time was ranked in the following trend: metal > plastic > classic printing. It could be explained by the sophistication associated with principle of metal 3D printing and its post‐processing processes—heat treatment, laser shock peening, laser polishing, and traditional machining. The post‐processing processes were essential to improve the mechanical and physical properties as well as surface characteristics of the products, albeit time‐consuming in nature [13]. Despite the similarity of the principles for metal and plastic printing—both utilized sintering technique for particle fusion and post‐processing for finalization, only support materials were needed as support for product for metal printing. Since manual removal of the support materials was subsequently undergone prior to the finalization of the 3D‐printed products thus it took the longest manufacturing time for metal printing. When it was compared with metal and plastic printing, the principle of classic printing was relatively simple as it only required the deposition of melted thermoplastics in a layer‐by‐layer fashion without post‐processing thus shortest manufacturing time with classic printing [14].

### 4.2 Preoperative Time and Operation Time

When preoperative time was considered, the following trend was observed: surgical guides > navigation system. Since the surgical guides were designed according to individual patients' tibial region, there were three factors taken into consideration—the anatomical structure of the tibial bone, the “anticipated” amount of correction in all axes and spatial location of the cutting plane which required more time when it was compared with the preoperative planning of navigation system which was mainly involved relatively simple procedures—from manual segmentation, 3D rendering, designing of cutting plane and introduction of surgical screws. However, the trend was different for operation time: navigation system > surgical guides. Prior to the commencement of navigation system‐assisted HTO, two important steps—five points and surface matching were undergone. A detailed guideline which was displayed on the screen of navigation system was cautiously followed to undergo the two types of matching technique which explained the relatively longer operation time with navigation system.

### 4.3 Accuracy

Surgical guides generated by metal 3D printing technique were the most accurate of all in terms of the angle deviation whereas guides generated by other techniques and navigation are relatively less accurate. It can be explained by several factors—rigidity, melting point, and accumulating error following each incision. First, the factor of rigidity—metal surgical guides were of the highest rigidity which prevented them from structural deformation and collapse during the cutting processes. However, plastic and classic surgical guides were of less rigidity which made them prone to be structurally damaged and deformed thus rendering lower accuracy. Second, Melting point– as the cutting continued, the temperature was increased to the melting points of the 3D printed surgical guides which resulted in structural deformities. Lastly, the error accompanying each incision—since the insertion of each pin into the bone model was not guided by a surgical guide, it gave rise to positional error which accumulated as the procedure proceeded and it resulted in the inaccuracy of navigation‐assisted system in HTO.

Although the findings were in favor of “surgical guides enabled more accurate corrections than navigation system,” there was lack of relevant scientific literature in the field of orthopedics that enable conclusive statements to be made. In other medical fields, the opinions towards surgical accuracy rendered by navigation system and surgical guides had been split into two major groups—one perceived that navigation system rendered higher accuracy in surgical setting than surgical guides and the other one perceived that there were no differences in accuracy rendered by either one of them. To cite a few, it was suggested by Dure et al. as the use of navigation system was more accurate than surgical guides in dental implant surgery. In the study, the positioning of 3D implant was carried out either with surgical guide (NobelGuide) or navigation system (Denacam). There was significantly lower degree of deviation in both angular and tip & mandibular regions with the use of Denacam whereas there was only significantly lower degree of deviation in depth and bucco‐oral angle with the use of NobelGuide [13, 14]. On the contrary, one literature held a neutral position in similar matter where they were assessing the deviations of preoperative planned position of the implant and postoperative implant position in three anatomical positions (coronal, apical, and angular deviations) to compare the accuracies of surgical guides and navigation system in dental implant surgery. The difference of deviations in all three positions was shown to be statistically insignificant thus implied that similar accuracies were achieved by surgical guides and navigation system [15].

### 4.4 Other Advantages With Surgical Guides

Other than high accuracy and shorter intra‐operative time [16], there were other advantages associated with the use of surgical guides in other types of surgeries. In the context of total knee arthroplasty (TKA) surgery, there was decrease in frequency of postoperative misalignment [17]. Significant differences between surgical guide and traditional surgical approaches in the number of cases with postoperative mechanical axis alignments within three degrees or even higher likelihood of alignment to almost zero value in “surgical guide” group were observed in multiple literatures [17, 18]. Second, practical simplicity‐ due to its simplicity in surgical application, it had contributed not only to reduction in inventory but also reduction in costs related to procedures such as sterilization and handling [19]. In the context of mandibular reconstruction surgery, the utilization of free fibular flaps has been the gold standard of this operation, but it has been associated with challenges for instance increase in ischemia time and reliance upon the experience of the surgeon which can be easily resolved with the use of surgical guides. In some literatures where surgical guides and traditional technique were employed, significant decrease in mean ischemia time of fibular osteoseptocutaneous flap was observed in “surgical guide” group which resulted in survival of fibular flap [19, 20, 21].

### 4.5 Limitations

There are several limitations with this study. First, a small sample size, also known as low statistical power, was associated with problems such as reduction in likelihood of obtaining true positives. It had been reported that studies with small sample size tend to obtain more false positives than expected [22]. Second, intra‐, and inter‐operator variability—during the bone model trials, more than one surgeon was carrying out the operations. Since there were potential differences and inconsistency in undertaking procedures between and within operators, it might influence the assessment of all the parameters when different types of surgical guides and navigation system were used and thus potentially contributed to unreliable and inaccurate results.

### 4.6 Future Investigations

In the prospective future, the following can be investigated: (1) augmented‐reality navigation system can be introduced to compare with surgical guides and navigation system upon the six parameters mentioned in this study and (2) also investigate other potential factors that might give rise to the findings other than the three aforementioned factors (melting point, rigidity, and accumulating error).

### 4.7 Prospects of Clinical Application

Metal 3D‐printed surgical guides are proving to be highly valuable in the clinical application of assisted HTO. By utilizing patients' medical imaging data, 3D printing technology can create metal guides that are highly customized to fit the individual anatomical structures of the patient, ensuring accurate positioning and cutting during the procedure. In clinical practice, these surgical guides enhance the precision of HTO, significantly reducing surgery time and minimizing the risk of postoperative complications. The strength and stability of metal materials provide robust support for recovery, while their biocompatibility helps decrease the likelihood of postoperative infections.

Looking ahead, the future of metal 3D‐printed surgical guides in HTO appears promising as 3D printing technology continues to advance. Researchers are exploring more efficient printing techniques and a wider range of materials to further enhance the functionality of these guides. Additionally, integrating artificial intelligence into surgical planning can lead to more intelligent, personalized treatment approaches.

Overall, the application of metal 3D‐printed surgical guides in assisted HTO not only improves the safety and effectiveness of the procedure but also opens new avenues for personalized medicine and precision treatment, indicating a broad developmental horizon for the orthopedic surgical field.

## 5 Conclusion

According to the findings, it was suggested that the metal 3D‐printing surgical guides were better suited for HTO surgeries than plastic, classic, and navigation system if accuracy and operation time were the only factors to be considered. It was speculated that three parameters (melting point, rigidity, and accumulative error) were responsible for the observed differences in correction accuracy. Further investigations were warranted to assess if other factors might influence these three parameters. To conclude, it was not recommended to use navigation system for assisting HTO given its inaccuracy in surgical incision and lengthened operation time.

## Author Contributions

Elvis Chun‐Sing Chui: conceptualization, data curation, methodology, software, formal analysis, writing original draft, final draft approval. Kyle Ka‐Kwan Mak, Randy Hin‐Ting Ng, and Ericsson Chun‐Hai Fung: methodology, software, formal analysis, writing – review and editing, final draft approval; Mei‐Shuen Chan, Kai Yue, Lawrence Chun‐Man Lau, Clifford Long‐Fung Chan, and Edmond Wing‐Fung Yau: data curation, resources, writing – review and editing, final draft approval. Wei Zhao, Xiuyun Su, Jin Zhang, and Jianglong Xu: validation, investigation, writing – review and editing, final draft approval. Hongxun Sang, Guoxian Pei, and Louis Wing‐Hoi Cheung: writing – review and editing, final draft approval. Sheung‐Wai Law and Michael Tim‐Yun Ong: conceptualization, methodology, resources, writing – review and editing, project administration, supervision, final draft approval. Patrick Shu‐Hang Yung: validation, writing – review and editing, final draft approval, funding acquisition.

## Disclosure

All authors had full access to all the data in this study and responsibility for the decision to submit for publication.

## Ethics Statement

The use of samples was approved by the Joint Chinese University of Hong Kong‐New Territories East Cluster Clinical Research Ethics Committee, Hong Kong.

## Consent

The authors have nothing to report.

## Conflicts of Interest

The authors declare no conflicts of interest.

## Data Availability

The datasets used and/or analyzed during this study are available from the corresponding author upon reasonable request.
